# Identifying Methamphetamine Abstainers With Convolutional Neural Networks and Short-Time Fourier Transform

**DOI:** 10.3389/fpsyg.2021.684001

**Published:** 2021-08-11

**Authors:** Xin Lai, Qiuping Huang, Jiang Xin, Hufei Yu, Jingxi Wen, Shucai Huang, Hao Zhang, Hongxian Shen, Yan Tang

**Affiliations:** ^1^School of Computer Science and Engineering, Central South University, Changsha, China; ^2^National Clinical Research Center for Mental Disorders, Department of Psychiatry, The Second Xiangya Hospital of Central South University, Changsha, China; ^3^Institute of Mental Health of Central South University, Chinese National Technology Institute on Mental Disorders, Hunan Key Laboratory of Psychiatry and Mental Health, Hunan Medical Center for Mental Health, Changsha, China; ^4^The Fourth People's Hospital of Wuhu, Wuhu, China

**Keywords:** methamphetamine abstainers, deep learning, short-time Fourier transform, functional magnetic resonance imaging, drug cues

## Abstract

Few studies have investigated the functional patterns of methamphetamine abstainers. A better understanding of the underlying neurobiological mechanism in the brains of methamphetamine abstainers will help to explain their abnormal behaviors. Forty-two male methamphetamine abstainers, currently in a long-term abstinence status (for at least 14 months), and 32 male healthy controls were recruited. All subjects underwent functional MRI while responding to drug-associated cues. This study proposes to combine a convolutional neural network with a short-time Fourier transform to identify different brain patterns between methamphetamine abstainers and controls. The short-time Fourier transformation provides time-localized frequency information, while the convolutional neural network extracts the structural features of the time–frequency spectrograms. The results showed that the classifier achieved a satisfactory performance (98.9% accuracy) and could extract robust brain voxel information. The highly discriminative power voxels were mainly concentrated in the left inferior orbital frontal gyrus, the bilateral postcentral gyri, and the bilateral paracentral lobules. This study provides a novel insight into the different functional patterns between methamphetamine abstainers and healthy controls. It also elucidates the pathological mechanism of methamphetamine abstainers from the view of time–frequency spectrograms.

## Introduction

Methamphetamine (MA) is a highly addictive stimulant with a continuously increased production and that is abused globally. MA addiction can lead to anxiety, depression, and psychosis (Zweben et al., 2004), causing structural and functional changes in the brain (Salo and Fassbender, 2012). Brain imaging provides valuable information on the neurobiological effects of drug abuse and helps explain the causes and mechanisms of vulnerability to drug abuse (Weinstein et al., 2016); however, there is limited neuroimaging research on the structural and functional recovery of the brain of long-term abstaining MA-dependent individuals. To overcome this barrier, we collected neuroimaging data from abstaining MA-dependent individuals (for at least 14 months) during the recovery period. Brain differences between healthy controls (HCs) and abstaining MA-dependent individuals were then studied to evaluate the risk of relapse and to understand the neurological impact of MA abuse.

Functional MRI (fMRI) is often used to study the functional characteristics of various brain regions in specific task states (Cassidy et al., 2018; Dvornek et al., 2018; Gui and Gui, 2020; Yousefnezhad et al., 2020; Yotsutsuji et al., 2021). Traditional statistical methods, such as the two-sample *t*-test, have played a significant role in locating abnormal brain regions associated with psychiatric disorders by looking at the brain activation levels during a task performance (Jiang et al., 2013). Usually, hypothesis testing may not fully capture the underlying group differences when there is a non-linear relationship (Smucny et al., 2021); hence, as an alternative, researchers have proposed to study neuronal oscillations or frequency responses associated with physiological functions (Han et al., 2011; Tang et al., 2016), where frequency analysis has been widely used to reveal the pathophysiology of mental diseases (Chang and Glover, 2010; Bolton and Van De Ville, 2019). These studies have highlighted the importance of studying the intrinsic brain activity within specific frequency bands for the resting-state fMRI. In this study, because long-term abstaining MA-dependent individuals show little difference from HCs using the traditional statistical methods, we propose to identify potential brain abnormality by exploring the spatiotemporal brain activation patterns within specific frequency bands. Short-time Fourier transform (STFT), a commonly used analysis method in physiological signal processing to extract information in the time–frequency domain (Sato et al., 2020), was used to study the changing spectra over time of MA-dependent individuals and HCs. STFT was used to analyze the dynamic changes of the frequency and phase information of a non-stationary signal (Subbarao and Samundiswary, 2016; Seeliger et al., 2018), which has many successful applications in pattern recognition, such as in speech (Takaki et al., 2019) and action (Klejmova and Pomenkova, 2017).

Regarding classifiers, we first resorted to traditional machine learning algorithms, such as support vector machine (SVM) and logistic regression (LR), which have shown excellent performance in the individual-level disease diagnosis (Pfister et al., 2011; Huang et al., 2015, 2016; Wang et al., 2015). However, we found it to be challenging to incorporate prior knowledge to extract biologically meaningful information from subtle changes, especially when it comes to features in the STFT spectrogram. Recently, deep convolutional neural networks (CNNs) have outperformed the traditional machine learning algorithms in capturing subtle changes in features in the network structure. CNN can extract non-linear network structures, can realize the approximation of complex functions, can characterize the distributed representation of input data, and can demonstrate the powerful ability to learn the critical features of datasets. It has been widely used in various fields, such as in the medical field (Fan et al., 2017). It is a very promising avenue offering better results compared with other conventional machine learning or statistical methods.

When granted the above considerations, we proposed an STFT-based CNN model to explore the abnormal brain regions in MA abstainers under the stimulation of drug-associated cues. Our model converted information on the brain area activation in fMRI into the STFT spectrograms, which were then fed into a CNN to generate the recognition results. Finally, we performed a 10-fold cross-validation to eliminate the interference of overfitting.

## Methods

### Participants

The study included 42 male MA-dependent individuals (aged 19–45 years) currently with a long-term abstinence status (at least 14 months) and 32 male HCs of similar age and education. Enrolment criteria for the two group is shown in Table 1. Exclusion criteria for all the subjects included (1) hallucinations, delusions, depression, anxiety, and other psychiatric symptoms; (2) a previous history of other axis I disorders (such as schizophrenia, depression, bipolar disorder, and mania); (3) a previous history of medical or physical therapy affecting brain functions, prescribed by psychiatry, neurology, or other specialties within the last 3 years; (4) a previous history of brain tumor, brain trauma, and other organic brain diseases; (5) a history of seizures, such as epilepsy, coma, high fever, and convulsions; (6) metabolic and endocrine diseases, cardiovascular diseases, and other physical diseases that affect brain functions; (7) metal or electromagnetic implants in the body, claustrophobia, and other conditions that are not suitable for the magnetic resonance examination; and (8) homosexuality. The detailed exclusion criteria are described in Chen et al. (2020).

**Table 1:** Enrolment criteria for MA abstainers and normal control groups.

**Variable**	**Controls (*n* = 32)**	**MA (*n* = 42)**	**p-value**
Age, y (SD)	33.58 (7.70)	32.67 (6.66)	0.542
Education, y (SD)	9.62 (2.25)	8.75 (2.12)	0.057
Duration of MA abstinence, mo (SD)	-	61.56 (37.71)	-
Age of first MA use, y (SD)	-	26.00 (6.97)	-
Tobacco use, y (SD)	13.09 (7.95)	15.90 (7.40)	0.051
Alcohol use, y (SD)	9.12 (5.94)	9.28 (5.60)	0.950

This study was approved by the ethical review board of the Second Xiangya Hospital of Central South University, which evaluated the study specifically related to the participation of individuals and conditions for the use of incarcerated individuals in research. Participants could decline their involvement in the study if they had any concerns, and all the participants provided voluntary written informed consent.

### Experimental Design and Procedures

A block design was adopted with a total of six sessions; each session contained one 20-s task block showing MA cue images and one 15-s rest block showing a crosshair. The MA cue images contained MA-related contents, e.g., people using MA, instruments used to consume MA, etc. Each block contained five unique images presented for 3 s with a 1-s interstimulus interval. Thus, a total of 30 images for each cue condition were presented during the fMRI scan. The images and blocks within sessions were randomly arranged.

### Image Acquisition and Preprocessing

All the fMRI scan images were collected at the Medical Imaging Department of the Second Xiangya Hospital of Central South University using a 3.0 Tesla Siemens MRI scan system. The subjects were required to lie flat on an examination bed equipped with a magnetic resonance scanner during the examination, wearing sponge earplugs and noise-proof headphones to reduce noise. An elastic sponge was used to fix the sides of the head and to reduce head movement. The fMRI data were synchronously collected while the subjects viewed the images. The parameters of magnetic resonance data acquisition were as follows: TR = 2000 ms, TE = 20 ms, the field-of-view (FOV) = 220 mm, matrix = 64 × 64, flip angle = 80°, voxel size = 3.4 × 3.4 × 3.4 mm^3^, slice thickness = 4 mm, and number of slices = 36. Interval scanning was employed, i.e., alternatively scanning the even- and odd-numbered layers. A total of 60 time points were collected.

DPARSF software (Chao-Gan and Yu-Feng, 2010) was used to preprocess the task fMRI data such as slicing time, head motion correction, spatial normalization with a 3 × 3 × 3 mm^3^ EPI template, and spatial smoothing with Gaussian kernel (FWHM = 6 mm). Participants met the standard by limiting their head motion within 2.5 mm. The number of displayed images of the drug-associated cues was 60.

### Development of the Discriminate Model

**Figure 1:**
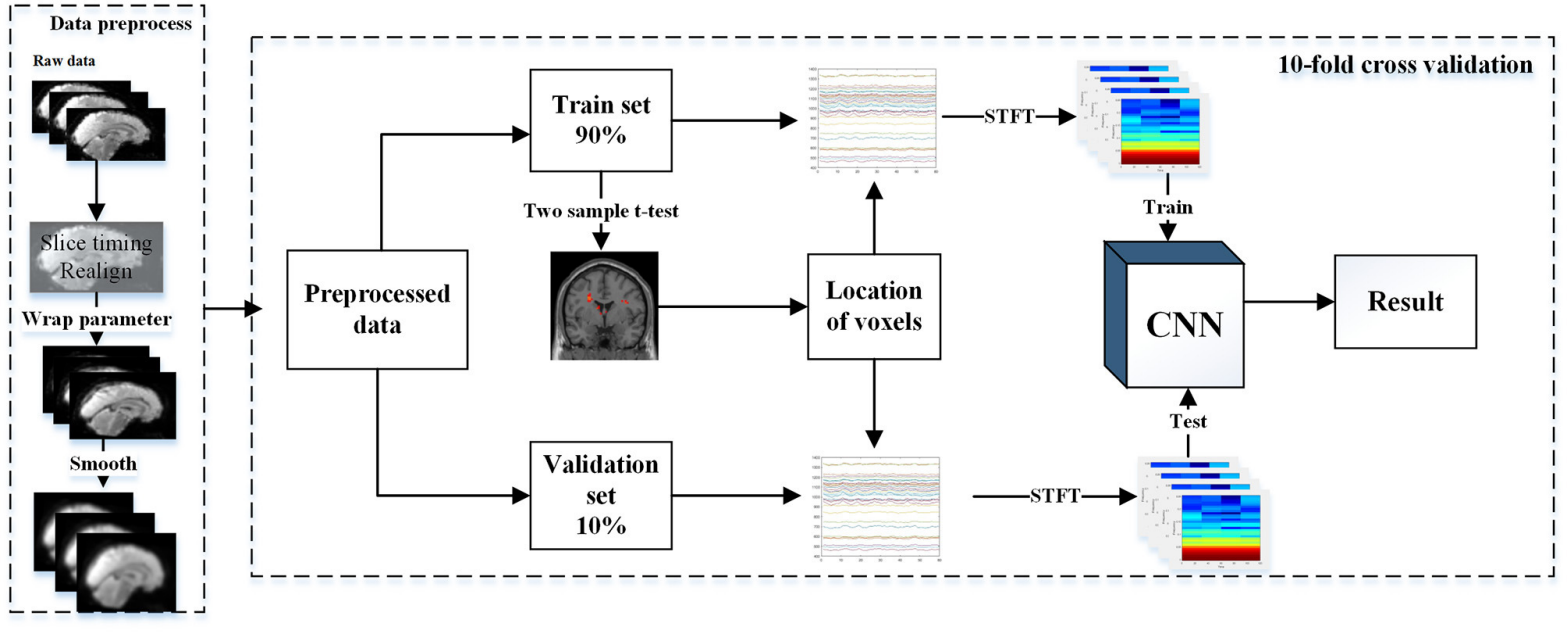
Model training and a 10-fold validation.

We divided the subjects into 10 groups, and 90% of the subjects were used for model training and the remaining 10% were used for model testing. We developed a data-driven classifier to further explore the group differences using a CNN model, which consists of four steps: feature selection, feature transformation, model identification, and cross-validation (Figure 1).

### Feature Selection

Considering the characteristics of huge voxels in the brain image, feature selection was used to obtain the locations of some significant voxels. To validate our algorithm, we only used the training data to build the generalized linear model (GLM) (first-level analysis). A fixed-effect boxcar waveform was convolved with the hemodynamic response function to produce a matrix to model categorical BOLD responses (Penny et al., 2003). In this procedure, a high-pass filter of 1/128 Hz was used to remove the low-frequency noise, and an AR (1) model was used to correct for temporal autocorrelations. To account for the residual motion artifacts, we included six motion regressors in our first-level model. Following the abovementioned procedure, neural activities associated with the drug cues were found. Thereafter, a two-sample *t*-test (Jiang et al., 2013) was conducted to compare abstaining MA-dependent individuals with HCs in the training data set to select the locations of voxels. When the two-sample *t*-test was used on the training data, there was no significant difference (*p* < 0.001 uncorrected) in the brain voxels. In this study, we only kept the locations of voxels with *t*-values greater than three and cluster sizes greater than five. The chosen number of voxels was denoted by ℕ, and we obtained the locations of these ℕ voxels through feature selection.

### Feature Transformation

Functional MRI time series is often performed by extracting the values of voxels after preprocessing. In this study, STFT was performed on the fMRI time series to investigate the time–frequency information for each subject using the following equation:

(1) $$\begin{array}{r} \text{STFT}\left(t,f\right)=\;\int_{-\infty }^{\infty }x\left(\tau \right)h\left(\tau -t\right)e^{-j2\pi f\tau }d\tau \end{array}$$

STFT divides a longer time signal into shorter segments of equal length (i.e., the size of window function *h*) and then computes the Fourier transform separately on each segment.

A previous study suggested a window size between 10 and 30 s to capture the dynamic information within the brain (Allen et al., 2014); however, owing to the limitation of the block size, we chose a window size of 10 s.

Here, based on the feature selection, STFT was performed on each ℕ voxel, giving us ℕ time–frequency spectrograms for each subject.

### CNN-Based Model

**Figure 2:**
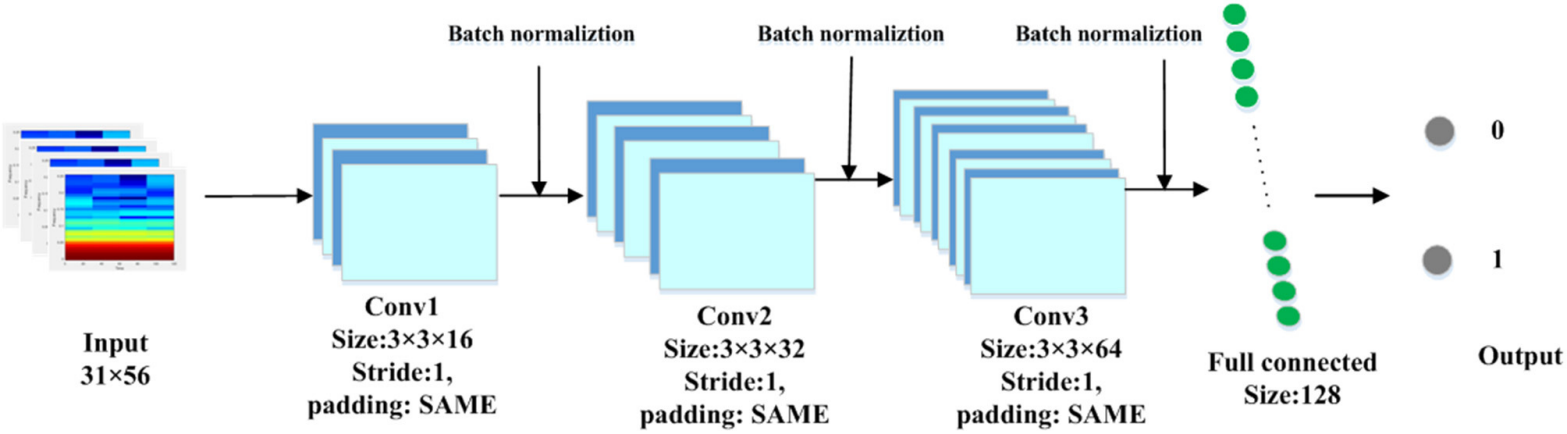
The network structure of convolutional neural network (CNN).

A CNN model is shown in Figure 2, which takes 31 × 56 time–frequency spectrograms as input and a two-element vector as output to classify a subject as abnormal or normal. The model includes three convolution layers with a rectified linear unit (ReLU) as the activation function, three batch normalization layers, and a fully connected layer. In all convolution layers, the kernel size was set to 3 and the padding mode was “SAME.” The stride of the convolution operation was set to 1. The kernel numbers of the three convolution layers were 16, 32, and 64, respectively, and each kernel corresponded to a feature map; thus, the feature maps of the three convolutional layers had sizes of 31 × 56 × 16, 31 × 56 × 32, and 31 × 56 × 64, respectively.

After each convolutional layer, we used the batch normalization layer to process the convolution result. Batch normalization was used to make the feature distribution more consistent with the real data distribution to improve the performance of the model. The activation functions introduced non-linearity to solve the deficiency of the expression ability of the linear model. We selected the ReLU as the activation function. Following the three convolutional layers, a fully connected layer with 128 neural units was used to merge the highly abstract features extracted by the convolutional layer to a softmax classifier. Finally, the softmax outputted two values that represented the probabilities of spectra belonging to the HCs and MA abstainers, respectively.

To train the CNN model, we used cross-entropy as the loss function, which can be formulated as given by Equation (2). Here, *y*_*i*_ represents the label of sample *i*, where “1” denotes MA abstainers and “0” denotes HCs; *p*_*i*_ represents the probability that sample *i* was predicted to be an MA abstainer.

(2) $$\begin{array}{r} L=-\left[y_{i}\cdot log\;\left(p_{i}\right)+\left(1-y_{i}\right)\cdot log\;\left(1-p_{i}\right)\right] \end{array}$$

### Cross-Validation

Because of the limited number of samples in this study, we used a cross-validation strategy to estimate the generalized performance of our classifier. Here, the performance of the classifier in the model for spectrograms was represented as *Acc*. We used permutation tests to assess the statistical significance of the cross-validation results. For permutation testing, the classification labels of the training data were randomly permuted 10,000 times. Cross-validation was then performed on every permuted training set. *Acc*_0_ was defined as the accuracy rate obtained by the classifier trained on the real class labels. When *Acc*_0_ exceeded the 95% (*P* < 0.05) CI of the classifier trained on the randomly relabeled class labels, it was assumed that the classifier had reliably learned the relationship between the data and the labels. For any value of the estimated *Acc*_0_, the *p*-value represented the probability of observing a classification prediction rate of no less than *Acc*_0_.

Considering that a single subject produced many time–frequency spectrograms probably belonging to different categories, we used Equation (3) to determine the category of a subject

(3) $$\begin{array}{r} Pre_{i}=\frac{T_{i}}{T_{i}+F_{i}}, \end{array}$$

where *Pre*_*i*_ represents the probability that subject *i* was successfully identified as an MA abstainer, *T*_*i*_ represents the number of time–frequency spectrograms for subjects that had been judged to be MA abstainers, and *F*_*i*_ represents the number of time–frequency spectrograms for subject *i* that were judged to be normal. When *Pre*_*i*_ was <0.5, we assumed that subject *i* was normal; otherwise, subject *i* was an MA abstainer. The average classification accuracies of the subjects were taken as the final result.

### Comparisons With Some Methods

To justify the effectiveness of the proposed method, some existing methods were tested on our dataset as comparisons. First, the traditional statistical method, a two-sample test, popular for data analysis in neuroimaging studies (Jiang et al., 2013), was compared with the proposed method. After data preprocessing, we divided all data into two groups (MA abstainers vs. matched controls) and used the two-sample test to identify the brain regions that showed the statistically significant MA-related differences (uncorrected *p* < 0.001). Second, considering that LR and SVM are widely used machine learning methods in psychiatry (Zhou et al., 2020), we used them as classifiers taking the STFT spectra as inputs. In the parameter setting of SVM, we set the penalty coefficient C to 1.0 and employed a Gaussian kernel function. In LR, we used L2 regularization with the penalty coefficient C equal to 1.0. We used the default values for other parameters. The classification accuracies of SVM and LR were reported after a 10-fold cross-validation.

## Results

### Classification Results

The detailed classification results of the cross-validation method used to verify the model recognition effect are shown in Table 2, where *Acc*_*CNN*_, *Acc*_*SVM*_, and *Acc*_*LR*_ represent the classification accuracy of spectrograms with CNN, SVM, and LR, respectively. We took the average of the model classification accuracy obtained during model verification. The average accuracy rate in our model was 93.4%, which was much better than that obtained using SVM and LR, whose average classification accuracies were only 76.2 and 72.0%, respectively.

**Table 2:** A 10-fold cross-validation accuracy of support vector machine (SVM) and convolutional neural network (CCN).

***Fold***	***Acc***_***CNN***_	***Acc***_***SVM***_	***Acc***_***LR***_	***Fold***	***Acc***_***CNN***_	***Acc***_***SVM***_	***Acc***_***LR***_
1	0.981	0.625	0.758	6	0.923	0.714	0.738
2	0.962	0.875	0.693	7	0.894	1.000	0.762
3	0.946	0.750	0.749	8	0.892	0.714	0.654
4	1.000	0.628	0.754	9	0.936	0.571	0.715
5	0.975	0.571	0.697	10	0.833	0.875	0.678

Permutation tests revealed that the proposed classifier learned the relationship between the data and the labels with an accuracy higher than 95%.

The recognition accuracy rate in our model, calculated using Equation (3) in “Cross-validation” section, ranged between 88.9 and 100% with an average value of 98.9%.

When we used the traditional statistical method (two-sample *t*-test) to detect the abnormal brain region, we could not find any statistically significant MA-related difference in this dataset.

### Brain Regions With a High Discriminative Power

Because the performance of the classifier was tested with a cross-validation strategy, the selected voxels might be different in separate iterations. The voxels that were included across all iterations were reported. Surprisingly, most of the voxels presented a clustered distribution. The difference distribution was mainly concentrated in the left inferior orbital frontal gyrus, the bilateral postcentral gyri, and the bilateral paracentral lobules. These areas mainly control the movements and emotions of the people.

## Discussion

**Figure 3:**
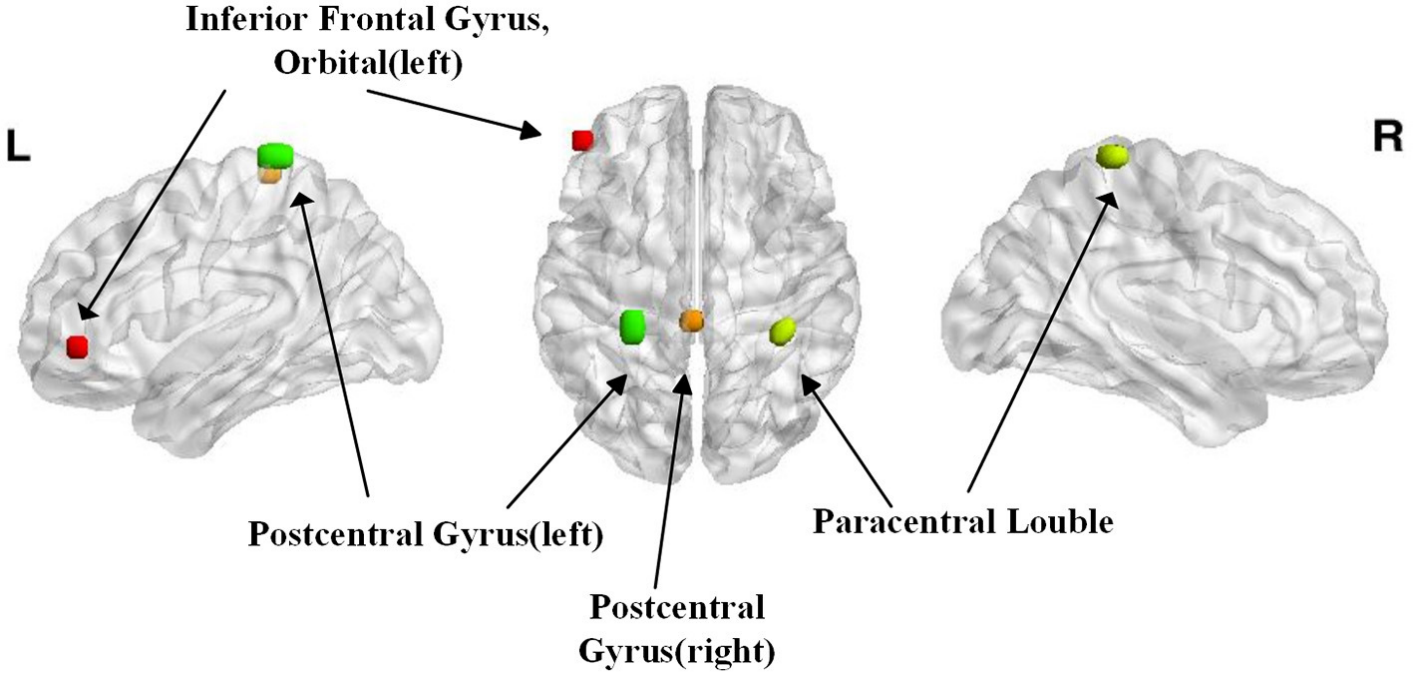
The regions with the highest discriminative powers in the Short-time Fourier transform (STFT)+CNN model.

In this study, we selected 42 MA abstainers and detected the differences in the brain activation regions when they saw drug cues. The result is shown in Figure 3. We achieved an average accuracy rate of 98.9% using STFT and CNN. The left inferior orbital frontal gyrus, the bilateral postcentral gyri, and the bilateral paracentral lobule gyri were associated with drug cues in MA abstainers.

Neuroimaging techniques have a high potential to detect brain deficits and correlations between the deficient brain regions and the cognitive–behavioral performance in MA abstainers. However, for MA abstainers, partial functions of the brain return to normal, and group-level statistical methods such as the two-sample test cannot detect any statistically significant MA-related difference in this dataset (Allen et al., 2014). However, using STFT and CNN, we found significant MA-related differences with the average accuracy rate reaching 98.9% in the cross-validation. The STFT method was used to analyze the time–frequency changes in the brain voxel signals. STFT is a time–frequency analysis technique suited to non-stationary signals and provides time-localized frequency information for situations in which the frequency components of a signal vary over time. Thus, this might be ascribed to the essentially non-linear neural dynamics of time–frequency changes underlying the brain activity. A similar conclusion was also obtained for MA abusers using electroencephalogram (EEG) (Khajehpour et al., 2019). In addition, without the pre-engineered features, CNN can conduct local perception and extract the spatial structural features of the time–frequency spectrograms. CNN methods have the potential to scale well and substantially improve the classification performance compared with SVM and LR methods (Abrol et al., 2021). Our findings highlight the presence of non-linearities and time–frequency changes in neuroimaging data that CNN can exploit as discriminative representations to characterize the MA abstainers.

Numerous MRI studies have documented that addictive drugs cause volume and tissue composition changes in the left inferior orbital frontal gyrus, which is associated with a longer duration of use of the MA (Volkow et al., 2015). Changes in the left inferior orbital frontal gyrus are likely associated with the cognitive and decision-making problems of the abusers. Moreover, this impairment of cognitive and altered decision-making in MA abstainers may result in relapse (Mizoguchi and Yamada, 2019), although this region may partially recover from long-term abstinence (Chang et al., 2005). In this study, we still detected different time–frequency spectrograms using the CNN model, i.e., MA abstainers were still influenced by the drug-associated cues, a deficit that is related to dysfunctions of the left inferior orbital frontal gyrus (Volkow et al., 2015).

The paracentral lobule controls the motor and sensory innervations, and the postcentral region is located in the somatosensory cortex. When these regions are impaired, the executive control systems may be affected as demonstrated by Volkow et al. (2015) by specific impairments within the executive brain networks in MA addicts during the exposure to drug-associated cues. Khajehpour et al. (2019) also reported that MA abusers differed in the gamma band in the paracentral lobule. It may be speculated that the substance-dependent individuals are unable to control their addiction-related behaviors (Khajehpour et al., 2019).

This study not only demonstrated a high classification accuracy of the STFT–CNN classifier from a drug–cue functional integration viewpoint but also elucidated the pathological mechanisms of the MA abstainers in a non-linear time–frequency characteristic. In the future, we will test this method on a larger independent dataset to confirm our findings.

## Data Availability Statement

The original contributions presented in the study are included in the article/supplementary material, further inquiries can be directed to the corresponding authors.

## Ethics Statement

The studies involving human participants were reviewed and approved by the ethical review board of the Second Xiangya Hospital of Central South University. The patients/participants provided their written informed consent to participate in this study.

## Author Contributions

XL, YT, and JX contributed to the conception of the study. XL, HY, JW, and JX designed the study. QH and SH collected the data. YT and XL performed data analysis and drafted the manuscript. HZ and HS modified the manuscript. All authors contributed to the article and approved the submitted version.

## Conflict of Interest

The authors declare that the research was conducted in the absence of any commercial or financial relationships that could be construed as a potential conflict of interest.

## Publisher's Note

All claims expressed in this article are solely those of the authors and do not necessarily represent those of their affiliated organizations, or those of the publisher, the editors and the reviewers. Any product that may be evaluated in this article, or claim that may be made by its manufacturer, is not guaranteed or endorsed by the publisher.
